# A comparison of authomatic vs. manual detection of anatomical landmarks during surface topography evaluation using the formtric 4D system

**DOI:** 10.1186/1748-7161-7-S1-O19

**Published:** 2012-01-27

**Authors:** P Knott, S Mardjetko, S Thompson

**Affiliations:** 1 Rosalind Franklin University, North Chicago, USA; 2 Illinois Bone and Joint Institute, USA

## Background

The Formetric 4D System (Diers International GmbH, Schlangenbad Germany) is a popular system for measuring surface topography in patients with adolescent scoliosis [1-4]. The system automatically detects anatomic landmarks on the patient, but then gives the user the opportunity to make adjustments to those landmarks if necessary. The purpose of this study was to see whether there would be more variability in repeated measurements if the landmarks were adjusted by the clinician or if they were left in the place where the machine had put them.

## Materials and methods

Twelve patients who had adolescent scoliosis of less than 30 degrees were measured for this study. Thirty repeated measurements of each patient were performed using the Formetric 4D, and the machine was allowed to select all the anatomic landmarks without assistance from the clinician. Each output parameter was analyzed to see the amount of variability that existed in the data. Each scan was then opened in the Formetric software, and the anatomic landmarks were adjusted by the clinician to move them to the exact location that coordinated with the visible surface topography. The data was then re-evaluated to see whether the amount of variability had increased or decreased.

## Results

Twelve parameters were compared, including the scoliosis angle. There were no statistically significant changes in any of the parameters before and after the landmarks were changed by the clinician.

## Conclusions

The conclusion is that it was not necessary for the clinician to make adjustments to the anatomic landmarks because the outcomes are not significantly changed by these manual adjustments.
